# Clinical Reports

**Published:** 1859-02

**Authors:** 


### CLINICAL REPORTS.

We commence in this number of the Journal "Reports" from the Clinic of the Atlanta Medical College, which we hope in the future, will constitute an interesting feature, in the original contributions to its pages. A *daily* Dispensary and Clinic has been established in the College, from which we are anticipating, not only a rich return to the Students of the Institution, but valuable aid to our Journal.

